# Multimedia Urban Road Path Optimization Based on Genetic Algorithm

**DOI:** 10.1155/2022/7898871

**Published:** 2022-04-30

**Authors:** Fangfang Ma

**Affiliations:** Foundational Courses Department, Shandong University of Science and Technology, Taian 271000, China

## Abstract

In order to study multimedia urban road path optimization based on genetic algorithm, a dynamic path optimization based on genetic algorithm is proposed. Firstly, for the current situation of traffic congestion, time constraints are strictly considered based on the traditional hard time window logistics distribution vehicle scheduling problem model. Then, the mathematical model is established, and the optimal solution is solved by the combination of decomposition coordination algorithm and genetic algorithm. We divide multiple customers into different customer groups and determine the service object order of each express car in each customer group, so as to obtain the most valuable scheduling scheme. Finally, in the process of solving the model, the relevant and reliable distribution basis for enterprise distribution is collected, including customer geographical coordinates, demand, delivery time window, unit cost required for loading and unloading, loading and unloading time, and penalty cost to be borne by distribution enterprises after early arrival and late arrival. Using the improved genetic algorithm, the optimal solution of each objective function is actually obtained in about 140 generations, which is faster than that before the improvement. Using the genetic algorithm based on sequence coding, a hybrid genetic algorithm is constructed to solve the model problem. Through the comparative analysis of experimental data, it is known that the algorithm has good performance, is a feasible algorithm to solve the VSP problem with time window, and can quickly obtain the vehicle routing scheduling scheme with reference value.

## 1. Introduction

**Figure 1:**
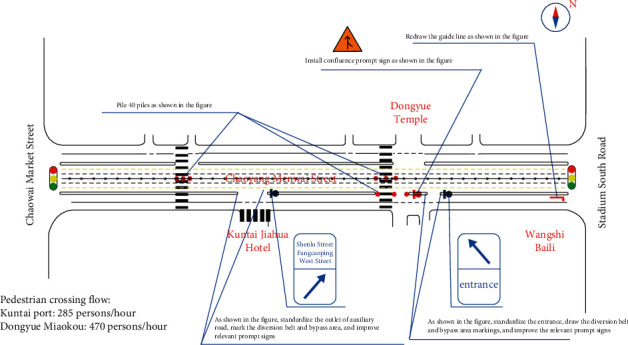
Optimizing urban road organization.

Transportation is closely related to human life. With the world economic and social progress and the acceleration of urbanization, transportation has gradually become the basis of national economic and social development and plays an important role in all aspects. However, with the increasing number of motor vehicles, frequent traffic congestion and even congestion, and the deteriorating traffic environment, these have become negative factors affecting social development. Accelerating the construction of transportation infrastructure and strengthening the management of urban transportation system are the two main ways to improve urban transportation [1]. Building roads is the most direct way to solve traffic problems. For example, building enough expressways can often solve the problem of traffic congestion between cities. Within cities, road construction must be carried out on the basis of the original urban road infrastructure, that is, the existing urban roads must be reconstructed. However, with the continuous increase of the number of urban motor vehicles and the continuous reduction of the space available for road construction in the city, only accelerating the construction of traffic infrastructure cannot effectively improve urban traffic. Therefore, within the city, we must integrate two ways (as shown in Figure 1), that is, while transforming the existing urban roads, we should constantly strengthen the R&D and management of the urban transportation system. In view of the current situation of traffic congestion, based on the traditional hard time window logistics distribution vehicle scheduling problem model, taking time as the primary factor affecting vehicle routing, this paper will establish a mathematical model and mix the decomposition coordination algorithm and genetic algorithm to solve the optimal solution [2]. Path optimization can help travelers find a suitable driving path, so as to reduce unnecessary stops of travelers on the road and finally realize the optimal distribution of traffic flow on each road section in the entire urban road network, which is very important for solving urban traffic congestion. Improving operating efficiency and driving safety factor, reducing energy consumption, and improving the traffic environment are of very positive significance. Genetic algorithm (GA) was proposed by J. Holland in 1975. GA is a highly parallel, random, and adaptive optimization algorithm based on “survival of the fittest.” It represents the solution of the problem as a “chromosome” survival of the fittest process. Through the continuous evolution of “chromosome” group from generation to generation, including replication, crossover, and mutation, it finally converges to the individual who is “most suitable for the environment,” so as to obtain the optimal solution or satisfactory solution of the problem.

## 2. Literature Review

Chen and others improved Dijkstra under two different conditions of allowing overtaking behavior and not allowing overtaking behavior, respectively. The improved algorithm allows the speed on a certain section to change at any time according to different sections, so it is more practical and representative than the simple Dijkstra algorithm. Aiming at the problem that the weight value of road section will change in the actual road network. D. Li and others proposed a new variable weight network optimal path algorithm combined with the road network model [4]. Sui and others proposed an unbalanced heuristic two-way A *∗* algorithm, which solves the problem of long search time of balanced heuristic two-way A *∗* algorithm and reduces the search space and improves the efficiency of path optimization algorithm. Aiming at the problem of long search time in complex road network [5]. Li and others introduced the parallel strategy, that is, divide the road network into multiple subnets, search in these subnets in parallel, and then connect the subnets through virtual nodes to finally obtain the optimal path of the whole road network [6]. Raman and others proposed an ant colony based path optimization algorithm for dual objective path optimization constraints [7]. Guo and others proposed a single source path optimization algorithm based on particle swarm optimization. Based on graph theory, the algorithm adopts a new coding rule to combine the local optimization mechanism with the periodic speed of particles to improve the path optimization efficiency of PSO [8]. Jiang and others put forward the problem of vehicle routing optimization, which connects the logistics and transportation problems of enterprises with operations research, and put forward the vehicle routing problem, which has attracted extensive attention. In practice, the vehicle routing problem can effectively improve the logistics and transportation efficiency of enterprises and also improve the efficiency of enterprises. Therefore, the vehicle routing problem has been widely concerned by academia and enterprises since it was put forward [9]. Zhang and others applied tabu search algorithm to vehicle routing optimization. The domain in the solution process of the algorithm is obtained through the tabu search process [10]. Cui and others used appropriate data structure to reduce the complexity of the problem. This method then became a good research basis for vehicle routing problem with time window [11]. Liang and others constructed an improved genetic algorithm to solve the vehicle routing optimization problem. This algorithm introduces a novel crossover operator, gets rid of the requirements for population diversity, and solves the common premature convergence problem of traditional genetic algorithm [12].

According to the current situation of traffic congestion, based on the traditional hard time window logistics distribution vehicle scheduling problem model, taking time as the primary factor affecting vehicle routing, this paper will establish a mathematical model and mix the decomposition coordination algorithm and genetic algorithm to solve the optimal solution. The central thesis of this article is as follows:

1. Analyze the traffic parameters and traffic operation status of urban roads and use fuzzy theory to convert the acquired real-time traffic parameters into a congestion degree with a value interval of [0, 1].
2. Understand the relevant knowledge of graph theory, design a dynamic road network model combined with the congestion degree, and give a mathematical model for dynamic path optimization.
3. Analyze and study the concept, principle, and characteristics of traditional shortest path algorithm and genetic algorithm and design a dynamic path optimization based on genetic algorithm combined with dynamic road network model.

## 3. Dynamic Road Network Model and Dynamic Optimal Path Model

Urban roads are born in response to the formation of cities. It changes with the development of the city. The road network is the hub connecting the city and transportation. Traffic network is the skeleton of a city and an important factor affecting urban development and urban traffic, and the traffic network model is the implementation basis of path optimization algorithm and various traffic simulation systems [13]. Therefore, it is very necessary to establish a traffic network model. In real life, urban traffic network has its own characteristics, mainly in the following aspects:

1. There are many nodes. There are usually thousands of road nodes in the traffic map of an ordinary city.
2. The length of road sections varies, and the angles and distances between roads are different.
3. The classification of roads is different. According to the specifications of China's road network, China's roads are mainly divided into expressway, trunk road, secondary trunk road, and branch road.
4. The congestion on the road section is not fixed but changes all the time with the change of real-time road conditions. That is, the congestion on the road section is different with the time of reaching the node. Even in the same section, the degree of road congestion is different at different time points, so the travel time of vehicles changes with time.

### 3.1. Static Road Network Model and Static Optimal Path Model

The traffic network is the basis for route optimization. Considering the intuitive structure of the network, it is most convenient and easy to represent the network with a diagram. Therefore, before studying the road network model, we first introduce some basic concepts in graph theory. According to the intuitive structure of the road network, the graph is the basic method to describe the road network, that is, the node of the road intersection correspondence graph, the road section between the two intersections corresponds to the edge in the graph, and the length of the road section is taken as the weight of the edge. According to the graph theory knowledge, the weighted graph can be used to describe the road network. In this way, solving the optimal driving route between two places can be transformed into solving the shortest distance between two points on the weighted graph.

However, the road network is a special network. Compared with the general network, it has its unique particularity, such as the attributes of nodes, edges, and so on. Next, the definitions of node, edge, and right of way in the static road network model will be given in combination with the actual traffic rules. Then, the road network is described in graph theory terms as follows:

(1) $$\begin{array}{c} D=\left\{d_{ij}|v_{i}\in V,v_{j}\in V\right\},\\ E=\left\{e_{1},e_{2},\ldots e_{m}\right\},\\ V=\left(v_{1},v_{2},\ldots v_{n}\right),\\ G=\left\{V,E,D\right\}, \end{array}$$

where *G* is the weighted directed graph representing the road network; *V*=(*v*_1_, *v*_2_,…, *v*_*n*_) is the set of road network nodes; *n* is the number of road network nodes; *E* is the set of road network edges; *m* is the number of road network edges; and *D* is the right of way matrix of the road network.

Because the actual length of the road is fixed within a certain period of time, the above road network model is called static road network model.

### 3.2. Dynamic Path Optimization Based on Genetic Algorithm

**Figure 2:**
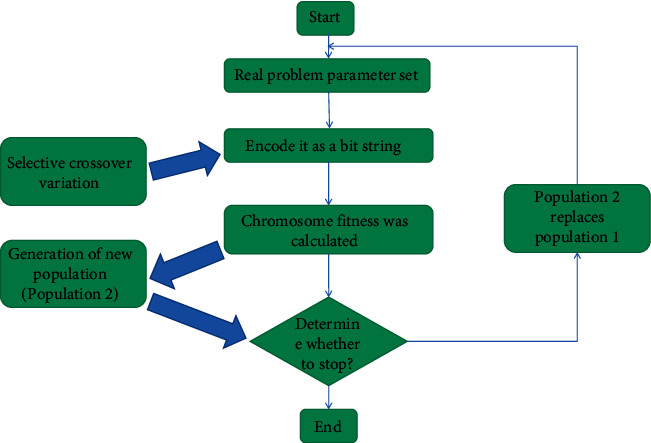
Working principle block diagram of basic genetic algorithm.

In the process of solving the problem, the solution space is regarded as the search space, and some possible solutions of the problem are encoded into chromosomes, which constitute a population [14]. Firstly, according to the optimization standard (fitness function), the chromosomes in the population are evaluated and the fitness value is calculated. According to the principle of “survival of the fittest” in the biological evolution theory, the chromosomes with large fitness are retained, the chromosomes with small fitness are eliminated, and the crossover and mutation are carried out with the help of genetic operators, so as to produce a new population until the predetermined optimization standard is reached. Then, the optimal chromosome in the final population is decoded to obtain the approximate optimal solution of the problem. It can be seen that genetic algorithm is a typical iterative algorithm. It starts from a group of randomly generated solutions. In each iterative process, it simulates biological evolution and genetic operation to generate a group of new solutions. The new solutions are evaluated by the fitness function, and the process is repeated until the algorithm reaches some form of convergence.  Figure 2 shows the working principle block diagram of the basic genetic algorithm.

As the first step of genetic algorithm design, the selection of coding method must fully consider the needs of practical problems and the influence of coding method on the design of genetic operators (especially crossover and mutation operators) [15].

For the path optimization problem of dynamic road network model, the following four points should be considered in the coding process:

1. The chromosome length is not fixed, that is, the number of points between the starting point and the ending point is unknown.
2. The genes in a chromosome cannot be duplicated, that is, the path cannot have a loop.
3. Coding should be easy to operate by crossover and mutation operators.
4. The coding shall cover all solutions in the problem domain.

At present, the common coding methods include binary coding, symbol coding, real coding, and floating point coding. In this paper, symbolic coding is used, that is, real numbers are used to represent nodes, and then these numbers are used as a coding form to participate in genetic operation. The coding method not only satisfies the above points but also simplifies the optimization procedure and ensures that the entire space where the global optimal solution may exist is searched.

The dynamic path optimization problem between two places in urban traffic can not be solved simply by the factor of distance, but a complex dynamic optimization process that integrates a variety of information such as road attributes and real-time traffic flow on the road [16]. Therefore, in the design of fitness function, the excellence of chromosome cannot be evaluated simply based on distance. Instead, all situations should be taken into account. According to the dynamic road network model and dynamic optimal path model, the dynamic right of way matrix *w*(*r*) can be expressed as

(2) $$\begin{array}{c} W\left(t\right)=\left\{W_{ij}\left(t\right)\right.=\left(\lambda_{1}+r_{ij}\left(t\right)+\lambda_{3}+1\right)d_{ij}|v_{i}\in V,\}|v_{j}\in V. \end{array}$$

The smaller the weight sum is, the lower the cost of the path is, that is, the higher the fitness of the chromosome is and the closer the corresponding possible solution is to the global optimal solution. Therefore, the fitness function in this paper is determined as follows:

(3) $$\begin{array}{c} f\left(x\right)=\frac{1}{\underset{\left(i,j\right)\in E}{\sum }w_{ij}\left(t\right)y_{ij}}, \end{array}$$

where *y*_*ij*_ is the decision variable. When chromosome *x* contains gene fragment (*i*, *j*) and the gene sequence remains unchanged, *y*_*ij*_=1; otherwise, *y*_*ij*_=0.

Genetic algorithm selects excellent individuals from the current population through selection operation and inherits them to the next generation, reflecting the principle of “survival of the fittest.” At present, the commonly used selection methods include sorting selection method, hierarchical selection method, and roulette selection method [17]. The basic idea of roulette selection method, also known as fitness proportional selection method, is that the probability of each chromosome being selected is directly proportional to its fitness value. When the population size is *N* and the fitness value of the *i*-th individual is *f*(*i*), the probability of the individual being selected is

(4) $$\begin{array}{c} p_{i}=\frac{f\left(i\right)}{\sum_{j=1}^{N}f\left(i\right)}. \end{array}$$

In order to solve the problems of “premature” and “convergence stagnation” easily caused by the roulette selection method, this paper adopts the fitness proportional selection method as the main selection method, combined with the optimal preservation strategy, so that the genetic algorithm can maintain a high level in both global and local search during the selection operation [18–20].

## 4. Application Example Analysis

### 4.1. Problem Description

**Figure 3:**
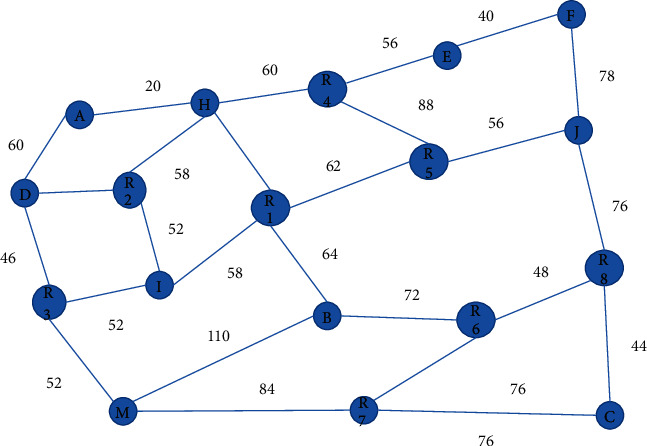
Road traffic situation in an area.

The express delivery center in a region has four express delivery vehicles of the same model and capacity, and the load capacity of each vehicle is 2 tons. Express items are now delivered to 60 customers in 9 regions of a city. The demand of each customer and the service time window required by customers are shown in Table 1, the distance between each customer point is shown in Table 2, and the road traffic conditions in this region are shown in Figure 3. For the convenience of calculation, the waiting time of express vehicles caused by traffic jam at different intersections is uniformly set. If there is a slight traffic jam, the waiting time is 10 min; in case of moderate traffic jam, the waiting time is 20 min; in case of severe traffic jam, the waiting time is 30 min. The driving speed of express vehicles is 80 km/h. Express vehicles start from the distribution center at 7:00 a.m. and return to the distribution center before getting off work at 18:00 p.m. At each customer location, it takes a total of 10 min to deliver the express at the customer location plus the waiting time. No matter how long the waiting time is caused by early arrival or late arrival, the cost is 20 yuan. It takes 10 min to reach different customer points in the same region. The peak of urban congestion is 7:00–9:00 in the morning and 17:00–18:00 in the afternoon. When there are multiple paths to reach a customer location, choose the path with the lowest cost and the shortest time [21].

### 4.2. Dispatching Scheme

This paper establishes a hard time window logistics scheduling model based on time and obtains the following vehicle scheduling paths.

Vehicle 1 route: start from the logistics center at 6:00 and return to the logistics center at 19:45.

B->45->47->2->5->7->26->27->30->31->34->37->38->35->33->28->32->B

Vehicle 2 route: start from the logistics center at 7:00 and return to the logistics center at 16:25.

B->52->54->53->50->19->22->20->1->3->4->6->46->48->49->B

Vehicle 3 route: start from the logistics center at 7:00 and return to the logistics center at 18:30.

B- >56->60->57->12->9->17->15->10->14->8->11->13->16->44->40->42->B

Vehicle 4 route: start from the logistics center at 5:00 and return to the logistics center at 19:55.

C- >25->29->36->39->41->43->51->55->59->58->18->21->23->24->B

### 4.3. Result Analysis

This paper uses C language to realize programming. In order to facilitate analysis and comparison, the simulation program is carried out in the same hardware environment.

It can be seen from Table 3 that the hybrid genetic algorithm is used to solve the example model for 10 times, and a high-quality solution is obtained. The average value of the total distribution time is 47.41 h, the average calculation time is 2.32 s, and the solution efficiency is high. For comparison, quantum ant colony algorithm [22] and improved genetic algorithm are used to solve the model algorithm. When the number of iterations is 200, the calculation results are shown in Table 4.

It can be seen from Table 4 that the efficiency and optimization structure of hybrid genetic algorithm are higher than those of quantum ant colony algorithm [23] and improved genetic algorithm.

**Figure 4:**
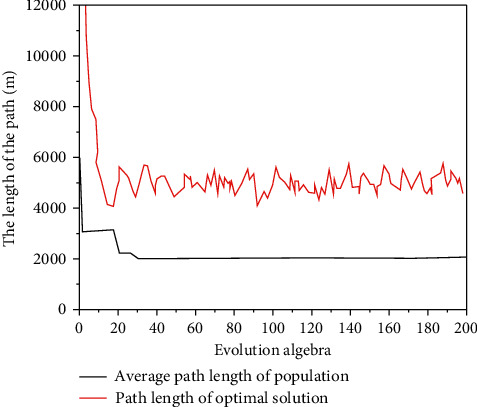
Variation law of population mean and optimal solution with iteration times in experiment 2.

Figure 4 shows the variation law of the mean fitness in the algorithm population and the number of iterations to obtain the optimal solution when the improved genetic algorithm solves the initial scheme of experiment 2 [24].

The change law of the mean fitness in the algorithm population shown in Figure 4 can be seen: while the algorithm retains the optimal solution, the improved crossover and mutation operation increases the diversity of the population, solves the “premature” problem of the basic genetic algorithm, and improves the global search ability of the algorithm [25]. It can be seen from the variation law of the number of iterations to obtain the optimal solution shown in the figure: for experiment 1, the algorithm converges to the optimal solution (4880 m) in about 50 generations; for experiment 2, the algorithm converges on the optimal solution in about 30 generations. Convergence to the optimal solution (1840 m), the improved genetic algorithm shows good convergence performance. The experiments verify the theoretical analysis of the convergence of the algorithm in this paper.

## 5. Conclusion

In this paper, the establishment of road network and dynamic path optimization algorithm is deeply studied, the real-time traffic flow information on the road is transformed into right of way, the concept, principle, and characteristics of traditional shortest path algorithm and genetic algorithm are analyzed and studied, the basic genetic algorithm is improved in combination with the dynamic road network model and the actual requirements for the path (eliminating the emergence of loop and open circuit), and a dynamic path optimization based on genetic algorithm is designed. The improved genetic algorithm is used to dynamically optimize the route. Using the dynamic route optimization algorithm, the determination method of the initial scheme of vehicle driving route and the adjustment method of dynamic driving route are formulated. In terms of algorithm improvement, this paper selects genetic algorithm to solve the multiobjective optimization problem, but the feasibility of large-scale customers is not specifically verified, and the gap between the optimal solution obtained by the improved algorithm and the real optimal solution is not confirmed. This needs further improvement and experiment. When establishing the mathematical model, this paper only considers the limitations of vehicle volume and the time constraints required by customers. However, in the actual distribution process, the vehicles in the distribution may be multiple models, and this paper is too single. Dynamic path optimization is becoming more and more important to urban traffic. With the popularity of intelligent vehicle terminals and smart phones, more and more people will use dynamic path optimization. It can be seen that for solving the problem of urban traffic congestion, using the urban traffic system that practices the dynamic path optimization algorithm has more advantages than speeding up the construction of traffic infrastructure.

## Figures and Tables

**Table 1:** Customer demand.

Region	Customer number	Demand (kg)	Earliest arrival time	Best earliest service time	Best latest service time	Latest arrival time
A	1	200	10:30	11:00	12:20	13:30
	2	300	8:30	9:00	9:30	10:00
	3	80	11:00	11:30	12:00	12:30

B	1	450	9:30	10:00	10:30	11:00
	2	700	8:00	8:30	9:00	9:30
	3	350	12:30	13:00	13:30	14:00

C	1	230	9:30	10:00	10:30	11:00
	2	360	14:00	14:30	15:00	15:30
	3	460	7:00	7:30	8:00	8:30

D	1	920	9:00	9:30	10:00	10:30
	2	70	7:30	8:00	8:30	9:00
	3	150	12:00	12:30	13:00	13:30

**Table 2:** Distance between regions and road conditions.

A	B	C	D	E	F	G	H
A			60				44
B	65			110	64	86	52
C		110					56
D	60		52				
E				52	64	40	20
F	52						110
G		40	64		58		
H		62				76	74

**Table 3:** Calculation results of hybrid genetic algorithm for example.

Calculation order	Number of vehicles used	Total delivery time (h)	The number of iterations to search the final solution for the first time	Calculation time (s)
1	4	50.3	97	2.30
2	4	51.23	132	2.30
3	4	47.56	185	2.30
4	4	46.19	167	2.30
5	4	49.58	121	2.30
6	4	42.53	149	2.30
7	4	44.69	163	2.30
8	4	42.98	139	2.30
9	4	48.63	175	2.30
10	4	47.03	143	2.30

**Table 4:** Comparison of calculation results of example genetic algorithm and hybrid genetic algorithm.

	Hybrid genetic algorithm	Improved genetic algorithm	Quantum ant colony algorithm
Average delivery time (h)	46.35	47.68	50.01
Standard deviation of solution	4.31	4.71	4.82
The final solution is found for the first time	147	153	161
Average calculation time (s)	2.16	2.36	2.57

## Data Availability

The data used to support the findings of this study are available from the corresponding author upon request.
